# Characterisation of a cell wall-anchored protein of *Staphylococcus saprophyticus *associated with linoleic acid resistance

**DOI:** 10.1186/1471-2180-12-8

**Published:** 2012-01-15

**Authors:** Nathan P King, Türkan Sakinç, Nouri L Ben Zakour, Makrina Totsika, Begoña Heras, Pavla Simerska, Mark Shepherd, Sören G Gatermann, Scott A Beatson, Mark A Schembri

**Affiliations:** 1 Australian Infectious Diseases Research Centre, School of Chemistry and Molecular Biosciences, The University of Queensland, Brisbane, QLD 4072, Australia; 2 Department of Medical Microbiology, Institute for Hygiene and Microbiology, Ruhr-Universität Bochum, Universitätsstr. 150, D-44780 Bochum, Germany; 3 Institute for Molecular Bioscience, The University of Queensland, Brisbane, QLD 4072, Australia; 4 Center for Infectious Diseases and Travel Medicine, University Medical Center Freiburg, Freiburg, Germany; 5 School of Biosciences, University of Kent, Canterbury, Kent CT2 7NJ, UK

## Abstract

**Background:**

The Gram-positive bacterium *Staphylococcus saprophyticus *is the second most frequent causative agent of community-acquired urinary tract infections (UTI), accounting for up to 20% of cases. A common feature of staphylococci is colonisation of the human skin. This involves survival against innate immune defenses including antibacterial unsaturated free fatty acids such as linoleic acid which act by disrupting bacterial cell membranes. Indeed, *S. saprophyticus *UTI is usually preceded by perineal skin colonisation.

**Results:**

In this study we identified a previously undescribed 73.5 kDa cell wall-anchored protein of *S. saprophyticus*, encoded on plasmid pSSAP2 of strain MS1146, which we termed *S. saprophyticus *surface protein F (SssF). The *sssF *gene is highly prevalent in *S. saprophyticus *clinical isolates and we demonstrate that the SssF protein is expressed at the cell surface. However, unlike all other characterised cell wall-anchored proteins of *S. saprophyticus*, we were unable to demonstrate a role for SssF in adhesion. SssF shares moderate sequence identity to a surface protein of *Staphylococcus aureus *(SasF) recently shown to be an important mediator of linoleic acid resistance. Using a heterologous complementation approach in a *S. aureus sasF *null genetic background, we demonstrate that SssF is associated with resistance to linoleic acid. We also show that *S. saprophyticus *strains lacking *sssF *are more sensitive to linoleic acid than those that possess it. Every staphylococcal genome sequenced to date encodes SssF and SasF homologues. Proteins in this family share similar predicted secondary structures consisting almost exclusively of α-helices in a probable coiled-coil formation.

**Conclusions:**

Our data indicate that SssF is a newly described and highly prevalent surface-localised protein of *S. saprophyticus *that contributes to resistance against the antibacterial effects of linoleic acid. SssF is a member of a protein family widely disseminated throughout the staphylococci.

## Background

Urinary tract infections (UTIs) are a universal source of human morbidity, with millions of cystitis and pyelonephritis episodes reported annually [1]. An estimated 40-50% of all women will experience at least one UTI in their lifetime, and one in three women will have had at least one clinically diagnosed UTI by the age of 24 [2]. Direct health care costs due to UTI exceed $1 billion each year in the USA alone [2]. *Staphylococcus saprophyticus*, a coagulase-negative staphylococcus, is the second most common causative agent of community-acquired urinary tract infection after *Escherichia coli *[3], and is responsible for up to 20% of cases. *S. saprophyticus *is of particular significance to sexually active young women, accounting for over 40% of UTI in this demographic [4]. *S. saprophyticus *UTI symptoms mirror those of *E. coli *[5] and recurrence is common, affecting 10-15% of infected women [6].

Three cell wall-anchored proteins, featuring a conserved characteristic C-terminal LPXTG motif, have previously been identified in *S. saprophyticus*. These proteins (i.e. SdrI, UafA and UafB) are all involved in adhesion [7-9], a crucial first step in the colonisation process. *S. saprophyticus *also possesses non-covalently surface-associated Aas [10,11] and Ssp [12] proteins that are implicated in virulence. Other than surface proteins, *S. saprophyticus *produces abundant urease which contributes to its ability to grow in urine [13]. Other putative virulence factors include cell surface hydrophobicity [14], slime [15] and D-serine deaminase [16].

Apart from rare complications, *S. saprophyticus *is only known to infect the urinary system [17-19]. The primary niches of this organism are in the human gastrointestinal and genitourinary tracts [4,20]. *S. saprophyticus *UTI is often preceded by colonisation of the perineal area; thus it can survive despite the innate immune defences of the skin. In this study, we have identified a previously undescribed LPXTG motif-containing cell wall-anchored protein of *S. saprophyticus*, termed SssF. The *sssF *gene is plasmid-encoded in *S. saprophyticus *strains ATCC 15305 and MS1146 and is highly prevalent in clinical isolates. We show that SssF belongs to a family of proteins conserved among staphylococcal species and contributes to survival against the staphylocidal free fatty acid linoleic acid - a component of the human innate immune defence system.

## Results

### Analysis of plasmid pSSAP2

**Figure 1:**
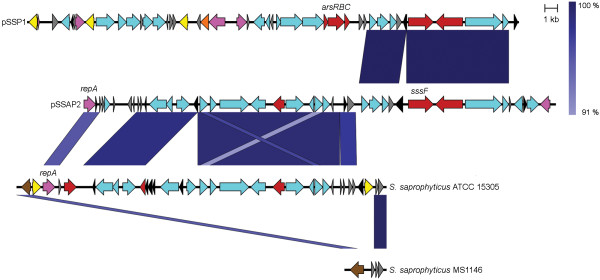
**Structure of the *S. saprophyticus *MS1146 plasmid pSSAP2 compared to the *S. saprophyticus *ATCC 15305 plasmid pSSP1, and the chromosomes of *S. saprophyticus *ATCC 15305 and *S. saprophyticus *MS1146**. Arrows represent CDS coloured according to their predicted function: no specific function (light blue); replication (pink); transposase for IS*431 *(yellow); other transposase (orange); integrase (brown); virulence-related (red); hypothetical protein (grey); and pseudogenes (black). Similarity regions between sequences are coloured in a gradient of blue, reflecting the percentage of nucleotide identity ranging from 91 to 100%, as illustrated on the scale on the top right of the figure.

*S. saprophyticus *strain MS1146, a clinical UTI isolate, has been described previously [7]. Its genome contains three plasmids - pSSAP1, pSSAP2 and pSSAP3. Sequence analysis of the 36 907 bp pSSAP2 plasmid revealed the presence of 35 predicted protein-coding genes, six pseudogenes and a mean G + C content of 29.9% (Figure 1 and Additional file 1: Table S1). Like other staphylococcal plasmids previously described, pSSAP2 has a mosaic structure with evidence of multiple insertions and deletions of discrete sequence blocks.

Plasmid pSSAP2 contains the *repA *gene and an approximately 17 kb region (from position 4 124 to 21 247) which share 96% and 97-99% nucleotide identity, respectively, with the chromosome of *S. saprophyticus *ATCC 15305 (Figure 1). A large proportion of the proteins encoded in this region are of unknown function or hypothetical, with the exception of a putative permease and several analogues of enzymes of the ribulose monophosphate pathway (Additional file 1: Table S1). Of note, the corresponding region in *S. saprophyticus *ATCC 15305 is longer (26 kb) and contains an arsenic resistance operon *arsRBC *and a putative lipase, both absent from pSSAP2. This region is also framed by two copies of the IS element IS*431*, which is frequently involved in the recombination-mediated integration of transposons and plasmids in methicillin-resistant *S. aureus *(MRSA) chromosomes [21,22]. Therefore, this region is likely to be an integrative plasmid of strain ATCC 15305; positioned upstream is a truncated integrase (SSP1642), for which an intact copy can be found in the *S. saprophyticus *MS1146 chromosome (Figure 1).

Another region of pSSAP2, ranging from position 21 529 to 33 235, shares ~99% nucleotide identity with plasmid pSSP1, which was originally described from *S. saprophyticus *ATCC 15305 [8]. The most notable feature of this region is the presence of a gene encoding for a LPXTG domain containing protein that we have designated *sssF *(see below).

### Sequence analysis of SssF staphylococcal homologues

**Figure 2:**
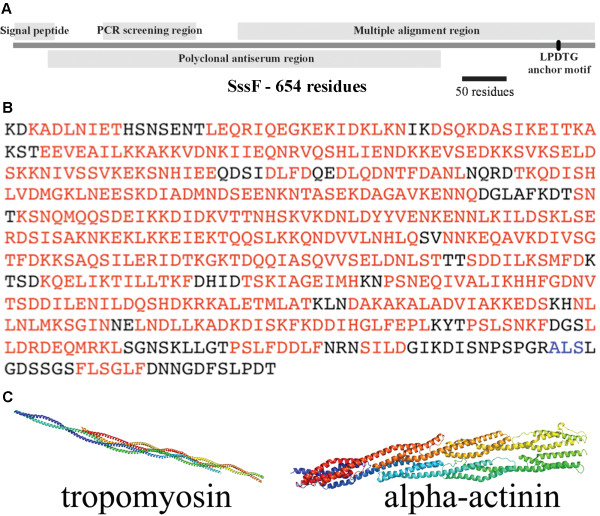
**Sequence analysis of SssF**. (**A**) Primary structure of the *S. saprophyticus *MS1146 SssF protein. The putative signal peptide, the corresponding gene region used for PCR screening, the region used in the multiple alignment (Additional file 2: Figure S1), the region used for polyclonal antibody raising and the LPDTG sortase anchor motif are indicated. (**B**) Structural prediction of the mature form of SssF. Residues coloured in red and in blue are predicted to adopt α-helical and β-strand conformations respectively. (**C**) Crystal structures of tropomyosin and alpha-actinin identified as likely structurally similar to SssF.

The *S. saprophyticus *MS1146 *sssF *gene is 1962 bp in length and the full-length translated SssF (*S. saprophyticus *surface protein F) protein contains 654 residues with a predicted molecular mass of 73.5 kDa (Figure 2A). SssF contains a predicted signal peptide of 45 residues (SignalP) [23] and an LPDTG anchor motif at the C terminus (Figure 2A), involved with covalent attachment of the mature protein to the cell wall. No conserved functional protein domains were detected, except for a possible albumin-binding GA module (Pfam PF01468, residues 58-109, E-value = 0.00039).

Sequence searches using the SssF amino acid sequence revealed similar proteins in other staphylococci. As expected, the SssF homologue encoded by pSSP1 in *S. saprophyticus *ATCC 15305 is near-identical at the protein level with only seven amino acid substitutions. Of note, every other sequenced staphylococcal genome contains an *sssF*-like gene, all chromosomally located except in *S. saprophyticus *(Additional file 2: Figure S1). Multiple alignment of the C-terminal regions (corresponding to the C-terminal 402 residues of SssF sequence) of one representative SssF-like protein from each sequenced staphylococcal species demonstrates there is variation from blocks of conserved and similar residues to regions of less similar sequence. This showed an overall protein identity ranging from 30.3-47.6%, versus *Staphylococcus pseudintermedius *HKU10-03 and *Staphylococcus carnosus *TM300, respectively, and an average amino acid identity of approximately 37% with the remaining SssF-like proteins. In terms of protein sequence similarity, these values range from 41.7% (*S. pseudintermedius *HKU10-03) to 84.4% (*S. carnosus *TM300). The N-terminal sequences are considerably more divergent.

All SssF-like proteins have a predicted signal peptide of between 35 and 45 residues, according to SignalP predictions. It is noted that the annotated *Staphylococcus haemolyticus *JCSC1435 SssF-like protein has an incorrectly called start codon, artifactually truncating the signal peptide sequence. All of the SssF-like proteins have a C-terminal sortase motif, implying cell surface localisation. Of the ten illustrated in Additional file 2: Figure S1, four have the canonical LPXTG motif, five have an alanine residue in the fourth position, and the *Staphylococcus lugdunensis *protein has a serine in this position.

### Structural prediction of SssF

Secondary structure predictions using PSI-PRED [24] indicate that SssF contains long, almost uninterrupted segments of α-helices (Figure 2B), which are likely to wrap around each other forming a rope-like coiled-coil structure. In order to predict its three-dimensional fold we carried out a fold-recognition analysis of SssF sequence using Phyre [25] (Protein Homology/AnalogY Recognition Engine). This server allows a pairwise alignment of the SssF sequence to a library of known protein structures available from the Structural Classification of Proteins (SCOP) [26] and the Protein Data Bank (PDB) [27] databases and generates preliminary models of the protein by mapping the sequence onto the atomic coordinates of different templates. Although SssF shares very low sequence identity with proteins in the PDB (range from 5-9%), this analysis identified several structural homologues of SssF with a confidence level of 100%. All the structures identified as likely analogues of SssF correspond to proteins that have a coiled-coil fold, including various types of the filamentous proteins such as tropomyosin [28] (PDB code: 1C1G) or alpha-actinin [29] (PDB code 1HCI) (Figure 2C), strongly suggesting that this protein shares a similar three-dimensional structure. Each of the SssF-like proteins (complete mature forms) of the other ten staphylococcal species indicated in Additional file 2: Figure S1 is also predicted to almost exclusively consist of α-helical coiled-coils with the same Phyre-predicted structural analogues as SssF (data not shown).

### The *sssF *gene is highly prevalent in *S. saprophyticus*

To assess the prevalence of *sssF *in *S. saprophyticus *we used PCR to screen our collections of clinical isolates originating from Australia, Germany and the USA. The *sssF *gene was detected in 84.6% (55/65) of Australian isolates, 90.9% (10/11) of American isolates and 88.3% (53/60) of German isolates.

### SssF is expressed at the *S. saprophyticus *cell surface

**Figure 3:**
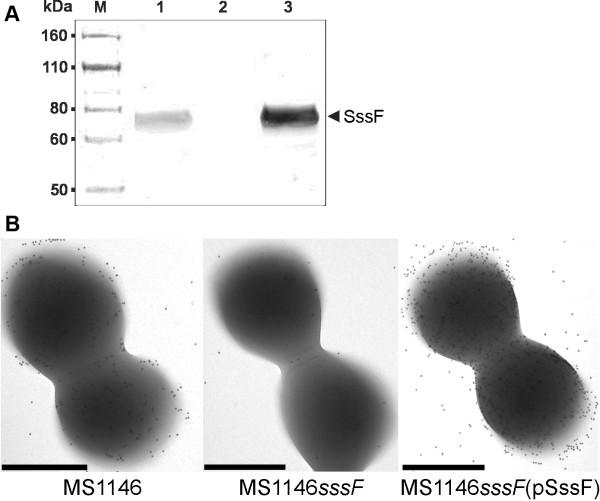
**Expression of SssF**. (**A**) Western blot analysis of whole-cell lysates prepared from *S. saprophyticus *MS1146, MS1146*sssF *and MS1146*sssF*(pSssF) using a polyclonal antiserum directed against SssF. Lanes: M, Novex Sharp Pre-stained protein marker (Invitrogen); 1, MS1146; 2, MS1146*sssF*; 3, MS1146*sssF*(pSssF). The position of SssF is indicated. Expression of SssF was detected in wild-type *S. saprophyticus *strain MS1146 and the *sssF *complemented strain but not in the isogenic *sssF *mutant. (**B**) Immunogold TEM of *S. saprophyticus *MS1146, MS1146*sssF *and MS1146*sssF*(pSssF). Expression of SssF at the cell surface of *S. saprophyticus *MS1146 was demonstrated by abundant labeling with SssF-gold particles. In contrast, *the sssF *isogenic knockout mutant was devoid of gold labeling. Complementation of the *sssF *mutation restored and enhanced surface expression of SssF. Bars, 500 nm.

In order to study the cellular localisation and function of the SssF protein, we generated an isogenic *S. saprophyticus *MS1146 *sssF *mutant (MS1146*sssF*) by insertional inactivation with a group II intron using the TargeTron system. We then complemented the *sssF *mutation by the introduction of a pPS44 staphylococcal vector containing the cloned *sssF *gene, to create MS1146*sssF*(pSssF). Western blot analysis of whole-cell lysates from *S. saprophyticus *MS1146, MS1146*sssF *and MS1146*sssF*(pSssF) using rabbit polyclonal anti-SssF serum raised against a recombinant truncated SssF protein, demonstrated expression of SssF in MS1146 but not MS1146*sssF*. Complementation of *sssF *restored SssF expression in MS1146*sssF*(pSssF) (Figure 3A). The anti-SssF serum was used in conjunction with immunogold labeling and electron microscopy to demonstrate localisation of the SssF protein at the cell surface. MS1146 and MS1146*sssF*(pSssF) exhibited abundant gold labeling whereas MS1146*sssF *was devoid of labeling (Figure 3B).

### SssF does not mediate adhesion to uroepithelial cells or colonisation of the mouse bladder

Initial investigations into the function of SssF found no evidence of adhesion (to T24 and 5637 human bladder carcinoma cells [American Type Culture Collection; ATCC], exfoliated human urothelial cells or a wide range of ECM and other molecules, including human serum albumin), invasion of 5637 bladder cells, cell surface hydrophobicity modulation, biofilm formation or serum resistance that could be attributable to SssF (data not shown). Strain MS1146 and derivatives colonised the mouse bladder in similar numbers in a mouse model of UTI (4.8-5.8 × 10^6 ^c.f.u. per 0.1 g bladder tissue), indicating that SssF does not contribute to colonisation in this infection model.

### *S. saprophyticus *strains containing the *sssF *gene are more resistant to linoleic acid than those lacking *sssF*

**Figure 4:**
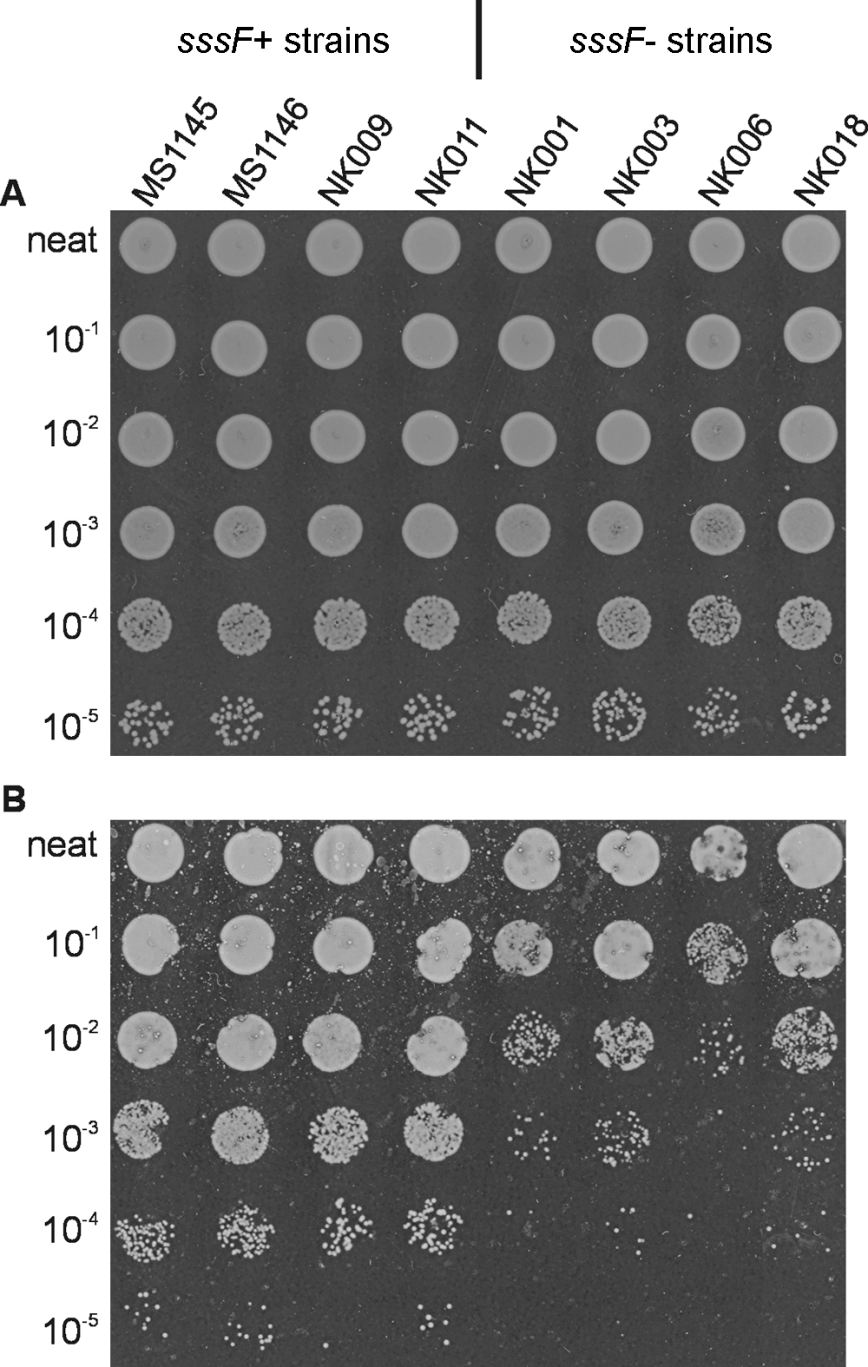
**Agar plate-based linoleic acid survival assay**. Relative survival of *sssF*^+ ^(including MS1146) and *sssF*^- ^*S. saprophyticus *strains on BHI agar medium supplemented with 0.85 M NaCl and containing 0 mM (**A**) or 5 mM (**B**) linoleic acid. The presence of the *sssF *gene is associated with increased (30-fold) resistance to linoleic acid. Serial dilutions of overnight *S. saprophyticus *cultures (2.5 μl) were spotted onto BHI agar + 0.85 M NaCl, containing 0 mM and 5 mM linoleic acid, 1% ethanol. The neat to 10^-5 ^dilutions are as indicated.

The above results prompted us to analyse the sequences of the family of SssF-like proteins to predict a function for SssF. The staphylococcal SssF-like proteins are all hypothetical proteins of unknown function except for SssF, which contributes to resistance of *S. aureus *to linoleic acid [30]. The mechanism of this phenotype remains unexplored. To determine whether SssF had a similar phenotype to the *S. aureus *SasF protein, linoleic acid survival assays were performed with *S. saprophyticus *MS1146 wild-type, MS1146*sssF *and MS1146*sssF*(pSssF) strains. No differences in survival among the strains were observed (data not shown). Following the lack of an observable phenotype for SssF in *S. saprophyticus *MS1146, we modified the linoleic acid emulsion assay to examine the survival of *S. saprophyticus *isolates that contain and do not contain the *sssF *gene in the presence of 0.85 M NaCl. Under these conditions, we observed a 30-fold difference in survival between the *sssF*^+ ^and *sssF*^- ^strains (*P *= 0.008; Figure 4). Using this modified protocol, we still observed no difference between the *S. saprophyticus *MS1146 wild-type and *sssF *mutant at linoleic acid concentrations of up to 25 mM (data not shown).

### SssF is associated with resistance to linoleic acid

**Figure 5:**
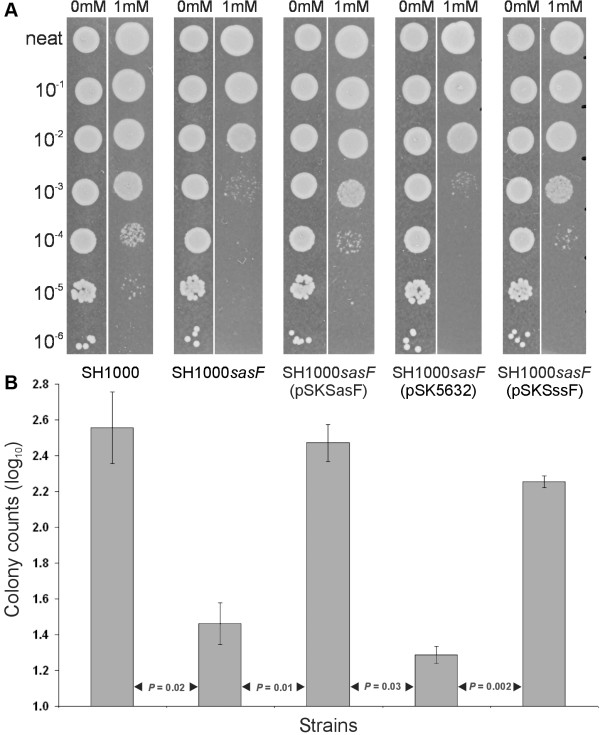
**SssF activity is detected in a *S. aureus *heterologous complementation approach**. (**A**) Relative survival of *S. aureus *SH1000 wild-type, SH1000*sasF *isogenic mutant and *sasF, sssF *and vector only complemented strains on agar medium containing 1 mM linoleic acid. Heterologous complementation of the *S. aureus *SH1000 *sasF *mutant with the *sssF *gene from *S. saprophyticus *MS1146 restores survival in these conditions. Serial dilutions of overnight *S. aureus *cultures (2.5 μl) were spotted onto BHI agar, pH 6.0, containing 0 mM and 1 mM linoleic acid, 1% ethanol. The neat to 10^-6 ^dilutions are as indicated. Shown are representative images from one of multiple experiments. (**B**) Graph showing the relative survival of *S. aureus *SH1000 and SH1000 derivates using data from Figure 5A. Colonies were counted after overnight incubation. Error bars represent ± SEM. Results from multiple experiments were analysed with Student's *t *test.

Survival assays were carried out with a *S. aureus *SH1000 genetic background, with the aim of determining if SssF could restore linoleic acid resistance of a *S. aureus *SH1000*sasF *knockout mutant (Figure 5). In agreement with a previous study [30], mutation of *sasF *in *S. aureus *SH1000 resulted in enhanced sensitivity to linoleic acid and this effect could be complemented by the introduction of a *sasF*-containing plasmid [SH1000*sasF*(pSKSasF)]. When the *sssF *gene from *S. saprophyticus *MS1146 was introduced into *S. aureus *SH1000*sasF*, resistance to linoleic acid was also restored, demonstrating that SssF contributes to the survival of *S. aureus *in the presence of linoleic acid.

## Discussion and conclusion

*S. saprophyticus *is a major cause of community-acquired UTI in young women. Knowledge of the virulence mechanisms of *S. saprophyticus *has advanced in recent years, particularly with the acquisition and analysis of whole genome sequence data. The majority of acknowledged virulence factors of *S. saprophyticus *are proteins tethered to the cell surface, which with the exception of the Ssp lipase [12], are all involved in adhesion: Aas is an autolysin that also binds to fibronectin [10]; UafA adheres to uroepithelial cells via an unidentified ligand [8]; SdrI binds to collagen I and fibronectin [9,31] and UafB binds to fibronectin, fibrinogen and urothelial cells [7]. Here we have identified another cell wall-anchored protein produced by *S. saprophyticus *that we have termed SssF - the sixth surface protein described for this species.

The *sssF *gene was identified in the sequence of the pSSAP2 plasmid of *S. saprophyticus *MS1146 due to the presence of the canonical LPXTG sortase motif in the translated protein sequence. A copy of the *sssF *gene is also located on the pSSP1 plasmid of *S. saprophyticus *ATCC 15305 (99% nucleotide identity; Figure 1), but it was not acknowledged as encoding an LPXTG motif-containing protein [8]. We recently characterised another plasmid-coded LPXTG motif-containing protein of *S. saprophyticus *MS1146, UafB, as an adhesin [7]. We first sought to investigate whether SssF was another adhesin, since a considerable proportion of characterised Gram-positive covalently surface anchored proteins have adhesive functions [32], including every other known *S. saprophyticus *LPXTG motif-containing protein. No evidence of an adhesion phenotype for SssF was detected.

SssF protein sequence searches with the BLAST database provided an output of uncharacterised staphylococcal proteins with a maximum 39% amino acid identity to SssF across the entire protein sequence, mostly annotated as hypothetical cell wall-anchored proteins. In contrast to *S. saprophyticus*, the genes encoding these SssF-like proteins are located on the chromosome, rather than on a plasmid, in every other sequenced staphylococcal species. Some of these staphylococcal SssF-like proteins contain atypical sortase motifs. At this stage it is not known whether all of these proteins are sorted to the cell surface efficiently, but SasF has been shown to be associated with the cell wall of *S. aureus *8325-4 even with the non-classical LPKAG sortase motif [33]. There was a distinct lack of phenotypic data for these SssF-like proteins until a role for SasF was recently uncovered. Kenny et al. [30] observed that *sasF *was the most upregulated gene in *S. aureus *MRSA252 microarray and qRT-PCR experiments upon challenge with linoleic acid. The protective function of SasF was apparent when examined in a linoleic acid emulsion agar plate-based bacterial survival assay. Our hypothesis focused on the possibility that SssF possessed a similar function to SasF, but no linoleic acid resistance phenotype for SssF was observed in the *S. saprophyticus *MS1146 genetic background. Using the linoleic acid emulsion agar plate bacterial survival assay in the presence 0.85 M NaCl, we observed a higher survival amongst *S. saprophyticus *strains that harbour the *sssF *gene than those that lack *sssF*. We then successfully expressed SssF heterologously in a *S. aureus *SH1000*sasF *host and demonstrated restored resistance to linoleic acid. We found *S. saprophyticus *MS1146 to be intrinsically more resistant to linoleic acid than *S. aureus *SH1000. This remains to be explored but could be due to a number of species/strain specific factors including the action of redundant *S. saprophyticus *MS1146 resistance mechanisms or variations in surface components such as capsule or teichoic acids.

We found that the survival of *S. aureus *SH1000 and its derivatives was markedly increased in the presence of linoleic acid at pH 6.0 compared to pH 7.4. This result is consistent with previous studies of the staphylococcal fatty acid modifying enzyme (FAME), an unidentified but partially characterised protein secreted by most staphylococci which detoxifies free fatty acids by esterifying them to an alcohol [34,35]. The FAME of *S. aureus *and *S. epidermidis *demonstrate optimal activity at pH 6.0, and have little activity at pH 7.4 [35,36]. This is congruent with human skin having a slightly acidic pH of 5.5-6 [37]. RP-HPLC experiments using linoleic acid and crude protein extracts demonstrated that SssF activity is distinct from FAME activity (data not shown). Other antimicrobial fatty acids such as sapienic acid have yet to be examined as substrates for SssF or SasF. We hypothesise that some or all of the other uncharacterised SssF-like proteins exhibit fatty acid resistance activity, but this remains to be demonstrated experimentally.

There are precedents for bacterial surface structures that provide protection against bactericidal free fatty acids. Gram-positive bacterial cell wall teichoic acids provide protection against free fatty acid mediated killing of *S. aureus *[38]. The IsdA protein of *S. aureus *reduces bacterial hydrophobicity when expressed at the cell surface under the cue of iron starvation to resist fatty acid membrane attack and also promotes fatty acid resistance of *S. aureus *in a volunteer human skin survival model [39]. Our studies however found that expression of SssF does not influence cell surface hydrophobicity of *S. saprophyticus*, and this corresponds with matching data for SasF and *S. aureus *[30].

No conserved motifs that might predict the functional residues of SssF-like proteins were identified. The observation that the SssF-like proteins are structurally related to myosin is noteworthy, especially in light of the recent characterisation of myosin cross-reactive antigens of *Streptococcus pyogenes *and *Bifidobacterium breve *as fatty acid hydratases [40,41]. These enzymes act to detoxify unsaturated free fatty acids, including linoleic acid. Homologous proteins with modest primary sequence identity but similar tertiary structures are acknowledged in both bacterial [42] and mammalian [43] lipid-binding protein families. It is possible that conserved tertiary protein structure between SssF-like proteins contributes to their function.

*S. saprophyticus *is a uropathogen, but SssF is unlikely to have evolved to facilitate survival in the urinary tract. A common trait of staphylococci is skin colonisation. Staphylocidal free fatty acids (especially unsaturated) are present on human skin [44] and are also active in staphylococcal abscesses [45]. Furthermore, linoleic acid is one of the most abundant polyunsaturated fatty acids on human skin [46], and is also present in vaginal secretions [47]. SssF may be an important determinant for survival of *S. saprophyticus *in the events preceding urethral entry in community-acquired UTI - colonisation of perineal and periurethral tissue. This would account for the absence of SssF involvement in the mouse model of UTI, in which the inocula are delivered directly into the bladder.

The location of *sssF *on a plasmid in both sequenced *S. saprophyticus *strains is intriguing, particularly as every other staphylococcal SssF-like protein is chromosomally encoded. It has been observed that many genes that are located on plasmids encode for traits which have extracellular functions [48], and *sssF *falls into this category. Furthermore, plasmid genes have often been noted to confer selective advantage to the bacteria in some environmental niches but not others [49]. Every pathogenic staphylococcal species known to carry a chromosomal *sssF*-like gene is known to commensally inhabit the skin, and this can be considered their main niche. *S. saprophyticus*, on the other hand, primarily resides in the genitourinary and gastrointestinal tracts [4,20]. It is feasible that since human skin is not the major habitat of *S. saprophyticus, sssF *has been retained as an accessory gene required for survival on the skin during non-UTI periods. Nonetheless, it may still be the case that *sssF *is found on the chromosome of some *S. saprophyticus *strains.

SssF represents the fourth LPXTG motif-containing protein described in *S. saprophyticus*. We present here evidence that the *S. saprophyticus *SssF protein has a role in the protection against free fatty acid mediated killing, and that it is a member of a newly identified protein family broadly distributed throughout the *Staphylococcus *genus.

## Materials and methods

### Bacterial strains and plasmids

The bacterial strains and plasmids used in this study are listed in Table 1. The clinical *S. saprophyticus *isolate collection used in this study is as previously described [7]. In addition, 60 clinical isolates from Germany were also tested. *S. saprophyticus *ATCC 15305 was described previously [8]. Staphylococcal strains were cultured in/on Brain Heart Infusion (BHI) broth/agar (Oxoid) supplemented with erythromycin or chloramphenicol (10 μg ml^-1^) as required. *E. coli *strains were cultivated in/on Luria-Bertani (LB) broth/agar supplemented with ampicillin (100 μg ml^-1^) as required.

**Table 1:** Strains and plasmids used in this study

Strain or plasmid	Description	Reference or source
***E. coli *strains**		

DH5α	F- φ80d*lacZ*ΔM15 Δ(*lacZYA*-*argF*)U169 *deoR recA1 endA1 hsdR17*(r_k_- m_k_+) *phoA supE44 *λ- *thi-1 gyrA96 relA1*	Grant *et al*. [50]

BL21	F- *ompT hsdS*_B_(r_B_- m_B_-) *gal dcm*	Stratagene

MS2066	DH5α containing pSssFHis	This study

MS2067	BL21 containing pSssFHis	This study

***S. saprophyticus *strains**		

ATCC 15305	Type strain (genome sequenced)	Kuroda *et al*. [8]

MS1146	Clinical isolate	AstraZeneca

MS1146*sssF*	MS1146 isogenic *sssF *mutant	This study

MS1146*sssF*(pSssF)	Complemented MS1146 *sssF *mutant	This study

***S. aureus *strains**		

SH1000	Functional *rsbU*-repaired derivative of *S. aureus *8325-4	Horsburgh *et al*. [51]

SH1000*sasF*	SH1000 isogenic *sasF *mutant	This study

SH1000*sasF*(pSKSasF)	SH1000 *sasF *mutant complemented with *sasF*	This study

SH1000*sasF*(pSKSssF)	SH1000 *sasF *mutant complemented with *sssF*	This study

SH1000*sasF*(pSK5632)	SH1000 *sasF *mutant with empty pSK5632 vector	This study

***S. carnosus *strains**		

TM300	Wild-type SK311	Schleifer & Fischer [52]

TM300(pSssF)	TM300 containing pSssF	This study

**Plasmids**		

pBAD/HisB	Cloning and protein expression vector, containing N-terminal 6 × His tag; Ap^r^	Invitrogen

pNL9164	*E. coli*/*S. aureus *TargeTron shuttle vector (temperature sensitive); Ap^r ^Em^r^	Sigma

pSK5632	Cloning and expression *E. coli*/*S. aureus *shuttle vector; Ap^r ^Cm^r^	Grkovic *et al*. [53]

pPS44	Staphylococcal vector, contains replicon and *cat *gene of pC194; Cm^r^	Wieland [54]

pSssFHis	1330 bp MS1146 *sssF *fragment, amplified with primers 873 and 874, digested with EcoRI/XhoI and cloned into EcoRI/XhoI-digested pBAD/HisB, with in-frame N-terminal 6 × His tag; Ap^r^	This study

pNK24	pNL9164 shuttle vector retargeted with primers 1001-1003, EBSU to knock out MS1146 *sssF *(TargeTron system); Ap^r ^Em^r^	This study

pNK41	pNL9164 shuttle vector retargeted with primers 2065-2067, EBSU to knock out SH1000 *sasF *(TargeTron system); Ap^r ^Em^r^	This study

pSKSssF	2394 bp fragment, including entire *sssF *gene from MS1146, amplified with primers 839 and 840 and cloned into the BamHI site of pSK5632; Ap^r ^Cm^r^	This study

pSssF	2400 bp BamHI/XbaI fragment, containing *sssF *gene, subcloned from pSKSssF into BamHI/XbaI-digested pPS44; Cm^r^	This study

pSKSasF	2175 bp fragment, including *sasF *gene from *S. aureus *NCTC 8325, amplified with primers 2084 and 2085 and cloned into the HindIII site of pSK5632; Ap^r ^Cm^r^	This study

### DNA manipulations and genetic techniques

Genomic and plasmid DNA were isolated as previously described [7]. PCR assays to determine the presence of *sssF *(primers 1127 and 1128) were performed using *Taq *DNA polymerase (NEB) under the following conditions: 2 min at 94°C, 25 cycles of 15 s at 94°C, 30 s at 55°C, 20 s at 72°C, 1 cycle of 3 min at 72°C, 4°C hold. Primers were synthesised by Sigma and are listed in Table 2. PCR amplification of the *sssF *gene was performed using Phusion Hot Start DNA Polymerase (Finnzymes).

**Table 2:** PCR primers used in this study

Primer	Sequence (5'-3')	Description
1127	GTTGAAGCAATATTGAAGAAAGC	*sssF *screen forward

1128	TTCTTCATTTAGTTTACCCATATCAAC	*sssF *screen reverse

839	GCTAGGATCCTCCATCTAATTCAAATGACAACG	*sssF *cloning forward. Contains BamHI site (underlined)

840	ACTAGGATCCGCTCCATTCAAAGTTCCACTTAC	*sssF *cloning reverse. Contains BamHI site (underlined)

873	GCTCACTCGAGTTCGACACCATCAGTAGAAGC	*sssF *fragment PCR for cloning into pBAD/HisB, for antibody production, forward. Contains XhoI site (underlined)

874	GCTCGGAATTCAAGCGCTTTAGCTTTAGCATC	*sssF *fragment PCR for cloning into pBAD/HisB, for antibody production, reverse. Contains EcoRI site (underlined)

1001	AAAAAAGCTTATAATTATCCTTAAGTCACTACTATGTGCGCCCAGATAGGGTG	*sssF *TargeTron IBS

1002	CAGATTGTACAAATGTGGTGATAACAGATAAGTCTACTATCTTAACTTACCTTTCTTTGT	*sssF *TargeTron EBS1d

1003	TGAACGCAAGTTTCTAATTTCGATTTGACTTCGATAGAGGAAAGTGTCT	*sssF *TargeTron EBS2

2065	AAAAAAGCTTATAATTATCCTTATCGTACGGCAAGGTGCGCCCAGATAGGGTG	*sasF *TargeTron IBS

2066	CAGATTGTACAAATGTGGTGATAACAGATAAGTCGGCAAGATTAACTTACCTTTCTTTGT	*sasF *TargeTron EBS1d

2067	TGAACGCAAGTTTCTAATTTCGGTTTACGATCGATAGAGGAAAGTGTCT	*sasF *TargeTron EBS2

2084	CAGTAAGCTTTGTTAGCGACATGGACAATATG	*sasF *cloning forward. Contains HindIII site (underlined)

2085	CCGTAAGCTTTTGCATATACTTCACAATAAATTAAGG	*sasF *cloning reverse. Contains HindIII site (underlined)

1011	TTCTTTAGGTGATGAACATATCAGG	Sequencing primer to check for correct 350 bp retargeted intron fragments for TargeTron

EBSU	CGAAATTAGAAACTTGCGTTCAGTAAAC	TargeTron EBS universal

### Bioinformatic analysis and identification of *sssF*

The *sssF *gene was identified in plasmid pSSAP2 of *S. saprophyticus *MS1146. The final pSSAP2 sequence was finished to Q40 standard with an average Sanger read depth of ~23 × coverage, which corresponds to an estimated number of four pSSAP2 plasmid copies per cell, based on the observed chromosomal read coverage (data not shown). Annotation of plasmid pSSAP2 was carried out manually using Artemis [55] and BLAST [56] similarity searches of publicly available sequence databases. The complete nucleotide sequence of *S. saprophyticus *plasmid pSSAP2 is available from the GenBank/EMBL/DDBJ database under accession number HE616681. The multiple alignment (Additional file 2: Figure S1) was created with CLUSTAL W2 [57] and edited with Jalview [58]. Figure 1 was produced using Easyfig [59].

### Construction and complementation of staphylococcal mutants

Plasmid construct pNK24 (Table 1), specifically retargeted to the *sssF *gene of *S. saprophyticus *MS1146, was prepared using the Sigma TargeTron Gene Knockout System, as per the manufacturer's instructions. Retargeting PCR primer sequences (1001-1003, Table 2) were determined by the TargeTron online design site, followed by a retargeting PCR and cloning of the PCR product into the provided shuttle vector, pNL9164 (Table 1). The construct was sequenced to verify correct inserts using primer 1011 (Table 2). The retargeted plasmid was then purified with a Qiagen Maxiprep kit and introduced into *S. saprophyticus *MS1146 by protoplast transformation as previously described [10], followed by CdCl_2 _induction and colony PCR screening to identify the *sssF *mutant (MS1146*sssF*). The *S. aureus *SH1000 *sasF *gene was also interrupted with the TargeTron system as above, using primers 2065-2067 (Table 2). The retargeted plasmid (pNK41, Table 1) was passaged through a restriction-deficient *S. aureus *strain (RN4220), then electroporated into *S. aureus *SH1000 and induced to create the *sasF *mutant (SH1000*sasF*). For complementation of the *S. saprophyticus *MS1146 *sssF *mutation, the *sssF *gene was initially amplified from *S. saprophyticus *MS1146 (primers 839 and 840, Table 2) and cloned into the BamHI site of pSK5632, forming plasmid pSKSssF. Plasmid pPS44 was digested with BamHI/XbaI and the vector part was ligated with the BamHI/XbaI *sssF*-containing fragment from pSKSssF to generate plasmid pSssF. Plasmid pSssF was used to transform *S. carnosus *TM300, re-isolated and then introduced into *S. saprophyticus *MS1146*sssF *by protoplast transformation. For complementation of the SH1000*sasF *mutation, *sasF *from *S. aureus *SH1000 was PCR amplified (primers 2084 and 2085, Table 2) and cloned into the HindIII site of pSK5632 to form plasmid pSKSasF, followed by electroporation of SH1000*sasF*. SH1000*sasF *was heterologously complemented with the *S. saprophyticus *MS1146 *sssF *gene by the introduction of pSKSssF. *S. aureus *SH1000*sasF *containing empty pSK5632 vector was also prepared as a control.

### Purification of truncated SssF, antibody production and immunoblotting

For antiserum production, a 1330 bp segment from *sssF *from *S. saprophyticus *MS1146 (Figure 2A) was amplified with primers 873 and 874 (Table 2), digested with XhoI/EcoRI and ligated into XhoI/EcoRI-digested pBAD/HisB. The resultant plasmid (pSssFHis) contained the base pairs 181-1510 of *sssF *fused to a 6 × His-encoding sequence. This *sssF *sequence corresponds to amino residues 39-481 of the SssF sequence. Protein induction and purification, inoculation of rabbits, staphylococcal cell lysate preparation and immunoblotting were performed as described previously [7], except NuPAGE Novex 4-12% Bis-Tris precast gels with NuPAGE MES SDS running buffer (Invitrogen) were used for the SDS-PAGE and *S. saprophyticus *MS1146*sssF*-adsorbed rabbit anti-SssF serum was used as the primary serum for the Western blot.

### Microscopy and image analysis

Immunogold labeling and transmission electron microscopy (TEM) were performed as described previously [7], using 1:10 anti-SssF serum as the primary antibody. No negative staining was performed.

### Linoleic acid survival assay

*S. aureus *linoleic acid survival assays were performed essentially as described by Kenny *et al*. [30]. Briefly, serial dilutions of overnight cultures (2.5 μl spots) were plated in duplicate onto BHI agar, pH 6.0, containing 0 mM or 1 mM linoleic acid. All agar media contained a final concentration of 1% ethanol. Colonies were counted after overnight incubation at 37°C. Mean values were compared using Student's *t *test. *S. saprophyticus *survival assays were performed similarly, but with agar plates containing 5 mM linoleic acid, supplemented with 0.85 M NaCl.

### Structural predictions of SssF

Secondary structure and three-dimensional fold predictions were performed using PSI-PRED [24] and Phyre [25], respectively.

## Authors' contributions

NPK identified the *sssF *gene, participated in the design of the study, performed sequence analysis, performed the preliminary SssF phenotypic experiments, performed the PCR prevalence screening, prepared the *sssF *antigen for antibody production, constructed the knockout mutants, performed the Western blots, prepared the samples for electron microscopy, performed the survival assays, and was the principal writer of the manuscript. TS performed the subcloning and transformations of *S. saprophyticus *and *S. carnosus *for the complementation of the *S. saprophyticus *MS1146 *sssF *mutant, and assisted in editing the manuscript. NLBZ prepared Figure 1 and Additional file 1: Table S1 and assisted in writing and editing the manuscript. MT performed the electron microscopy and assisted in editing the manuscript. BH performed the structural predictions of SssF and prepared Figure 2B and 2C. PS participated in the RP-HPLC and assisted in editing the manuscript. MS participated in the RP-HPLC and assisted in editing the manuscript. SGG provided the German *sssF *prevalence data and assisted in editing the manuscript. SAB co-directed the research and assisted in writing and editing the manuscript. MAS directed the research and assisted in writing and editing the manuscript. All authors read and approved the final manuscript.

## Supplementary Material

Additional file 1 **Table S1**. Predicted protein-coding genes of pSSAP2. Click here for file

Additional file 2 **Figure S1**. ClustalW alignment of the C-terminal 402 amino acid residues of *S. saprophyticus *MS1146 SssF protein (61% of entire sequence) with corresponding sequence from other staphylococcal SssF-like proteins, showing clusters of amino acid conservation. Only one representative protein from each species is shown. Sequences are sorted (in descending order) by similarity to *S. saprophyticus *MS1146 SssF sequence, which ranges from 31.1% (*S. pseudintermedius *HKU10-03) to 48.5% (*S. carnosus *TM300). Jalview was used to colour-code the alignment by percentage identity. The C-terminal sortase anchor motifs are indicated by a red box. GenBank accessions for the SssF-like proteins are as follows: *S. carnosus *TM300, CAL29334; *S. capitis *SK14, EEE48467; *S. caprae *C87, EFS16450; *S. epidermidis *RP62A, AAW53125; *S. warneri *L37603, EEQ79103; *S. haemolyticus *JCSC1435, BAE03665; *S. hominis *SK119, EEK11979; *S. aureus *NCTC 8325, ABD31969; *S. lugdunensis *HKU09-01, ADC86449; *S. pseudintermedius *HKU10-03, ADV06726. Click here for file
