# Supplementary material for: Characterisation of a cell wall-anchored protein of Staphylococcus saprophyticus associated with linoleic acid resistance
Source: BMC Microbiol. 2012 Jan 15;12:8. doi: 10.1186/1471-2180-12-8 (PMC3398289; doi:10.1186/1471-2180-12-8)
Supplement: Additional file 1 — Table S1. Predicted protein-coding genes of pSSAP2. [file 1471-2180-12-8-S1.DOC]

**Table S1.** Predicted protein-coding genes of pSSAP2

| **Feature id^1^** | **Start^2^** | **End** | **Strand** | **Description** |
| --- | --- | --- | --- | --- |
| *repA* (SSAP_P201) | 1 | 938 | + | replication initiator protein A |
| SSAP_P202 | 914 | 1051 | + | replication protein (pseudogene) |
| SSAP_P203 | 1236 | 1334 | + | hypothetical protein |
| SSAP_P204 | 1432 | 1599 | + | hypothetical protein |
| SSAP_P205 | 1621 | 2049 | + | putative small heat shock protein |
| SSAP_P206 | 2316 | 2429 | + | hypothetical protein |
| SSAP_P207 | 3457 | 3732 | - | hypothetical protein |
| SSAP_P208 | 3857 | 3943 | - | hypothetical protein |
| SSAP_P209 | 4236 | 4331 | + | hypothetical protein |
| SSAP_P210 | 4442 | 4549 | + | hypothetical protein |
| SSAP_P211 | 4760 | 5101 | - | glutathione peroxidase (pseudogene) |
| SSAP_P212 | 5123 | 6466 | - | putative dehydrogenase |
| SSAP_P213 | 6453 | 6911 | - | transcriptional regulator |
| SSAP_P214 | 7257 | 8270 | + | putative alcohol dehydrogenase |
| SSAP_P215 | 8598 | 8912 | - | arsenate reductase (pseudogene) |
| SSAP_P216 | 9075 | 9716 | + | putative hexulose-6-phosphate synthase |
| SSAP_P217 | 9872 | 10390 | + | ThiJ/PfpI family protein |
| SSAP_P218 | 10649 | 12910 | + | excinuclease ATPase subunit |
| SSAP_P219 | 13052 | 14200 | - | alcohol dehydrogenase |
| SSAP_P220 | 14772 | 15683 | - | putative permease |
| SSAP_P221 | 15809 | 17221 | + | putative transcriptional regulator |
| SSAP_P222 | 17627 | 17971 | - | putative transcriptional regulator |
| SSAP_P223 | 18062 | 18694 | + | putative hexulose-6-phosphate synthase |
| SSAP_P224 | 18694 | 19245 | + | putative 6-phospho-3-hexuloisomerase |
| SSAP_P225 | 19344 | 19493 | - | hypothetical protein |
| SSAP_P226 | 20093 | 20546 | + | hypothetical protein (pseudogene) |
| SSAP_P227 | 20646 | 21170 | + | conserved hypothetical protein |
| SSAP_P228 | 21739 | 22290 | + | putative site-specific recombinase |
| SSAP_P229 | 22339 | 23226 | + | putative transcriptional regulator |
| SSAP_P230 | 23336 | 23860 | + | putative acyl-CoA hydrolase |
| SSAP_P231 | 23868 | 24242 | + | hypothetical protein |
| SSAP_P232 | 24367 | 24939 | - | dehydrogenase (pseudogene) |
| *sssF* (SSAP_P233) | 25371 | 27335 | + | *S. saprophyticus* surface protein F |
| SSAP_P234 | 27501 | 29618 | - | N-acetylmuramoyl-L-alanine amidase |
| SSAP_P235 | 29837 | 32686 | + | hypothetical protein, similar to phage infection protein |
| SSAP_P236 | 32772 | 33227 | + | transcriptional regulator |
| SSAP_P237 | 33270 | 33419 | + | hypothetical protein |
| SSAP_P238 | 33658 | 34296 | - | putative metal dependent phosphohydrolase |
| SSAP_P239 | 34396 | 34635 | + | bacterial luciferase family protein (pseudogene) |
| SSAP_P240 | 34652 | 35584 | + | zinc-binding oxidoreductase |
| SSAP_P241 | 35691 | 36461 | - | replication-associated protein |

^1^The locus tag for the *S. saprophyticus* MS1146 genome project is SSAP. ^2^Start and end positions relative to the pSSAP2 sequence.
