# Supplementary figures and images for: Characterisation of a cell wall-anchored protein of Staphylococcus saprophyticus associated with linoleic acid resistance
Source: BMC Microbiol. 2012 Jan 15;12:8. doi: 10.1186/1471-2180-12-8 (PMC3398289; doi:10.1186/1471-2180-12-8)

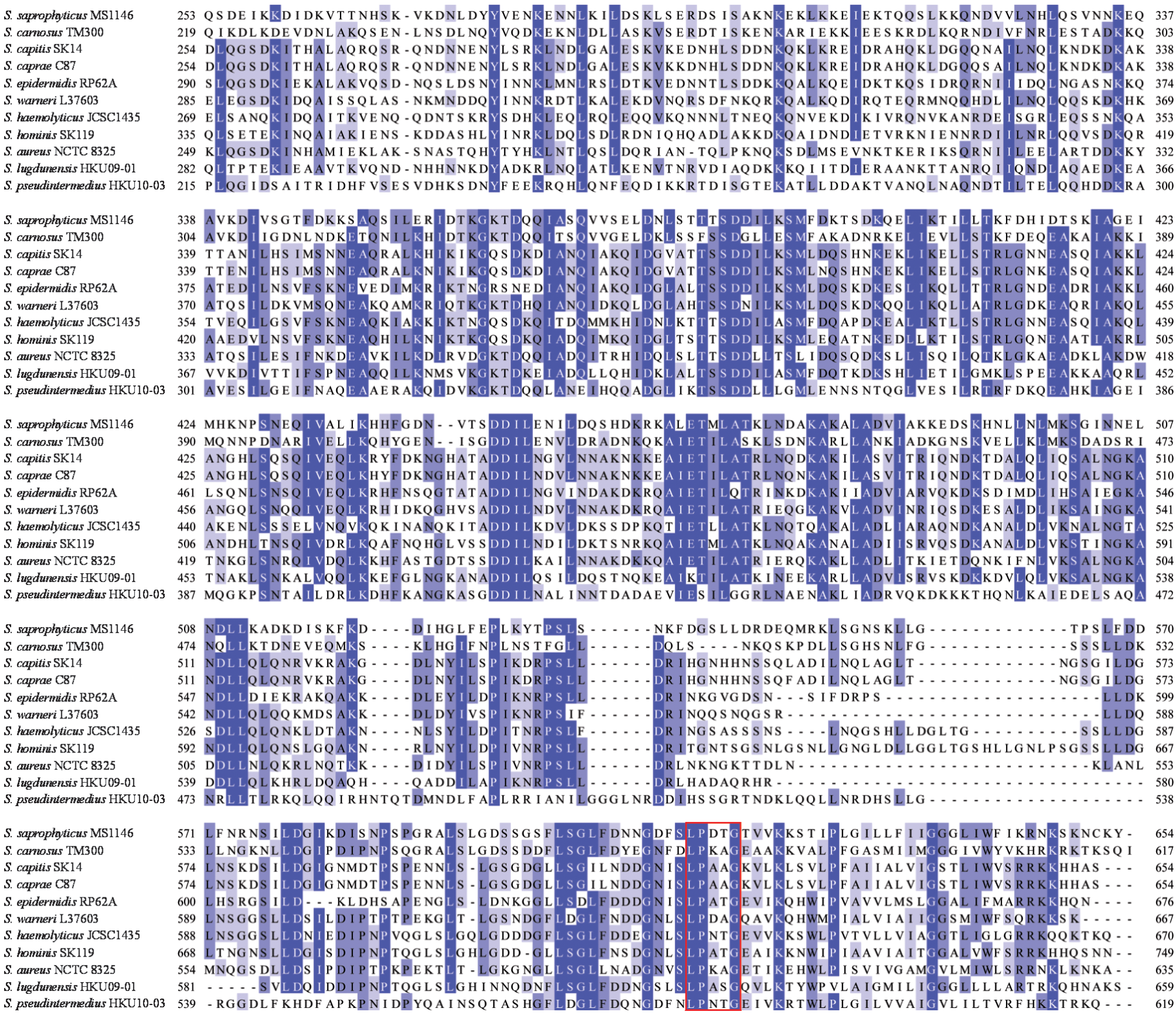

Supplement: Additional file 2 — Figure S1. ClustalW alignment of the C-terminal 402 amino acid residues of S. saprophyticus MS1146 SssF protein (61% of entire sequence) with corresponding sequence from other staphylococcal SssF-like proteins, showing clusters of amino acid conservation. Only one representative protein from each species is shown. Sequences are sorted (in descending order) by similarity to S. saprophyticus MS1146 SssF sequence, which ranges from 31.1% (S. pseudintermedius HKU10-03) to 48.5% (S. carnosus TM300). Jalview was used to colour-code the alignment by percentage identity. The C-terminal sortase anchor motifs are indicated by a red box. GenBank accessions for the SssF-like proteins are as follows: S. carnosus TM300, CAL29334; S. capitis SK14, EEE48467; S. caprae C87, EFS16450; S. epidermidis RP62A, AAW53125; S. warneri L37603, EEQ79103; S. haemolyticus JCSC1435, BAE03665; S. hominis SK119, EEK11979; S. aureus NCTC 8325, ABD31969; S. lugdunensis HKU09-01, ADC86449; S. pseudintermedius HKU10-03, ADV06726. [file 1471-2180-12-8-S2.TIFF]
